# Tips and Tricks and Clinical Outcome of Cryopreserved Human Amniotic Membrane Application for the Management of Medication-Related Osteonecrosis of the Jaw (MRONJ): A Pilot Study

**DOI:** 10.3389/fbioe.2022.936074

**Published:** 2022-07-22

**Authors:** Stéphane Odet, Christophe Meyer, Camille Gaudet, Elise Weber, Julie Quenot, Stéphane Derruau, Sebastien Laurence, Lisa Bompy, Marine Girodon, Brice Chatelain, Cédric Mauprivez, Esteban Brenet, Halima Kerdjoudj, Narcisse Zwetyenga, Philippe Marchetti, Anne-Sophie Hatzfeld, David Toubeau, Fabienne Pouthier, Xavier Lafarge, Heinz Redl, Mathilde Fenelon, Jean-Christophe Fricain, Roberta Di Pietro, Charlotte Ledouble, Thomas Gualdi, Anne-Laure Parmentier, Aurélien Louvrier, Florelle Gindraux

**Affiliations:** ^1^ Service de chirurgie maxillo-faciale, stomatologie et odontologie hospitalière, CHU Besançon, Besançon, France; ^2^ Laboratoire de Nanomédecine, Imagerie, Thérapeutique EA 4662, Université Bourgogne Franche-Comté, Besançon, France; ^3^ Pôle Médecine bucco-dentaire, Hôpital Maison Blanche, CHU Reims, Reims, France; ^4^ Université de Reims Champagne-Ardenne, Laboratoire BioSpecT EA-7506, UFR de Pharmacie, Reims, France; ^5^ Université de Reims Champagne Ardenne, Biomatériaux et Inflammation en Site Osseux, Pôle Santé, URCA, HERVI EA3801, UFR de Médecine, Reims, France; ^6^ Chirurgie Maxillo-Faciale - Stomatologie - Chirurgie Plastique Réparatrice et Esthétique - Chirurgie de la main, CHU de Dijon, Dijon, France; ^7^ Université de Reims Champagne Ardenne, Biomatériaux et Inflammation en Site Osseux, Pôle Santé, URCA, BIOS EA 4691, Reims, France; ^8^ UFR d’Odontologie, Université de Reims Champagne Ardenne, Reims, France; ^9^ Service d’ORL et chirurgie cervico-faciale, CHU Reims, Reims, France; ^10^ Banque de Tissus CBP CHU Lille, Lille, France; ^11^ Institut de Cancérologie ONCOLILLE CANTHER, UMR9020 CNRS–U1277 Inserm—Université de Lille, Lille, France; ^12^ Banque de Tissus, CHU Rouen, Rouen, France; ^13^ Activité d’Ingénierie Cellulaire et Tissulaire (AICT), Établissement Français du Sang Bourgogne Franche-Comté, Besançon, France; ^14^ Université Bourgogne Franche-Comté, INSERM, EFS BFC, UMR1098, RIGHT Interactions Greffon-Hôte-Tumeur/Ingénierie Cellulaire et Génique, Besançon, France; ^15^ Établissement Français du Sang Nouvelle-Aquitaine, Bordeaux, France; ^16^ INSERM U1035, Université de Bordeaux, Biothérapie des Maladies Génétiques Inflammatoires et Cancers (BMGIC), Bordeaux, France; ^17^ Ludwig Boltzmann Institute for Experimental and Clinical Traumatology/AUVA, Research Center, Vienna, Austria; ^18^ Austrian Cluster for Tissue Regeneration, Vienna, Austria; ^19^ Univ. Bordeaux, INSERM, BIOTIS, U1026, Bordeaux, France; ^20^ CHU Bordeaux, Service de chirurgie orale, Bordeaux, France; ^21^ Department of Medicine and Ageing Sciences, Gabriele D’Annunzio University of Chieti-Pescara, Chieti, Italy; ^22^ StemTeCh Group, Gabriele D’Annunzio Foundation, University of Chieti-Pescara, Chieti, Italy; ^23^ Inserm CIC 1431, CHU Besançon, Besançon, France

**Keywords:** human amniotic membrane, osteonecrosis, oral mucosa, allograft, bisphosphonates, denosumab, antiangiogenic drugs

## Abstract

Medication-related osteonecrosis of the jaw (MRONJ) is a complication of certain pharmacological treatments such as bisphosphonates, denosumab, and angiogenesis inhibitors. There are currently no guidelines on its management, particularly in advanced stages. The human amniotic membrane (hAM) has low immunogenicity and exerts anti-inflammatory, antifibrotic, antimicrobial, antiviral, and analgesic effects. It is a source of stem cells and growth factors promoting tissue regeneration. hAM acts as an anatomical barrier with suitable mechanical properties (permeability, stability, elasticity, flexibility, and resorbability) to prevent the proliferation of fibrous tissue and promote early neovascularization at the surgical site. In oral surgery, hAM stimulates healing and facilitates the proliferation and differentiation of epithelial cells in the oral mucosa and therefore its regeneration. We proposed using cryopreserved hAM to eight patients suffering from cancer (11 lesions) with stage 2–3 MRONJ on a compassionate use basis. A collagen sponge was added in some cases to facilitate hAM grafting. One or three hAMs were applied and one patient had a reapplication. Three patients had complete closure of the surgical site with proper epithelialization at 2 weeks, and two of them maintained it until the last follow-up. At 1 week after surgery, three patients had partial wound dehiscence with partial healing 3 months later and two patients had complete wound dehiscence. hAM reapplication led to complete healing. All patients remained asymptomatic with excellent immediate significant pain relief, no infections, and a truly positive impact on the patients’ quality of life. No adverse events occurred. At 6 months of follow-up, 80% of lesions had complete or partial wound healing (30 and 50%, respectively), while 62.5% of patients were in stage 3. Radiological evaluations found that 85.7% of patients had stable bone lesions (*n* = 5) or new bone formation (*n* = 1). One patient had a worsening MRONJ but remained asymptomatic. One patient did not attend his follow-up radiological examination. For the first time, this prospective pilot study extensively illustrates both the handling and surgical application of hAM in MRONJ, its possible association with a collagen sponge scaffold, its outcome at the site, the application of multiple hAM patches at the same time, and its reapplication.

## 1 Introduction

The human amniotic membrane (hAM) is the innermost layer of fetal membranes. It is composed of a single layer of epithelial cells, a basement membrane, and an avascular stroma, underlayered by the chorion. The thickness of hAM varies among individuals and depends on the location of the sample (70–180 μm thick) (Chen et al., 2012; Gremare et al., 2019). hAM is not homogeneous (Centurione et al., 2018). It contains amniotic epithelial cells and amniotic mesenchymal stromal cells (Parolini et al., 2008) and variable quantities of growth factors (Russo et al., 2011; Mcquilling et al., 2017). Basic preservation methods for hAM are cryopreservation, lyophilization, and storage in a dry form (Jirsova and Jones, 2017). Cell survival after cryopreservation, despite the addition of cryoprotective agents, is questionable (Laurent et al., 2014b).

The beneficial effects of hAM have been widely described in the literature. It is a biocompatible scaffold with suitable mechanical properties (permeability, stability, elasticity, flexibility, resorbability, and transparency) (Chen et al., 2012; Fenelon et al., 2021). Additionally, it possesses antifibrotic (Ricci et al., 2013), antiscarring (Mamede et al., 2012), antimicrobial (Chen et al., 2019), anti-inflammatory (Bailo et al., 2004; Wolbank et al., 2007), and analgesic properties (Rama et al., 2001; Dua et al., 2004; Gajiwala and Gajiwala, 2004). It modulates angiogenesis, having both pro- and antiangiogenic properties (Mamede et al., 2012; Gholipourmalekabadi et al., 2019), and induces epithelialization and wound healing (Mamede et al., 2012; Gholipourmalekabadi et al., 2019). Finally, it has low immunogenicity (Kubo et al., 2001), which makes it suitable as an allograft.

To date, ophthalmology is one of the most popular hAM indications in routine use to treat ocular surface diseases, including conjunctival surface reconstruction, corneal surface reconstruction, and as a substrate for the *ex vivo* expansion of limbal and conjunctival stem cells (Mamede et al., 2012). Since the mid-1990s, there has been a growing interest in using hAM for oral surgery to accelerate tissue regeneration. Two systematic reviews of literature explored the different indications for hAM use in this specific field (Fenelon et al., 2018; Odet et al., 2021), highlighting two types of application as “implanted graft material” or “covering graft material.” The first one applies to gingival recession, bone furcation defects, bone defects in interproximal areas, and surgical wounds after implant surgery; the second involves mandibular vestibuloplasty or mucosal defects.

Medication-related osteonecrosis of the jaw (MRONJ) is a complication caused by various treatments including bone antiresorptive agents (bisphosphonates and denosumab) and angiogenesis inhibitors (sunitinib, bevacizumab, among others). Clinically, symptoms can not only include pain, infection (often relapsing) with pus discharge, halitosis, and bone exposure but also serious local, regional (cellulitis, osteitis, and oro-cutaneal fistulas), or systemic infections and bone fractures. MRONJ is divided into four stages: zero (no bone exposure, only nonspecific clinical signs), one (asymptomatic bone exposure), two (bone exposure with pain or local infection), and three (symptomatic bone exposure with local, regional, or systemic infection, bone fracture, and orocutaneous fistulas). Following the American Association of Oral and Maxillofacial Surgeons (AAOMS) 2014 recommendations, conservative treatment is needed for stages 0–2, while surgical treatment is needed only for stage 2 MRONJ resistant to conservative treatments and for stage 3 MRONJ. There is currently no consensus on the management of MRONJ.

One case report and one case-control study (Ragazzo et al., 2018; Ragazzo et al., 2021) explored the use of hAM in 26 patients affected by MRONJ. They showed promising results in terms of wound healing, clinical outcomes, and significantly improved quality of life and pain, measured on a visual analog scale (VAS). The limits of the case-control study are the mixing of 1) stages (from 1a to 3, based on the Italian Society for Oral Pathology and Medicine/Italian Society for Maxillofacial Surgery (SIPMO/SICMF) classification), 2) diagnoses (cancer and osteoporosis), and 3) the route of drug administration (per os, intravenous (IV) and subcutaneous) and type of drugs (bone antiresorptive agents and angiogenesis inhibitors) that probably impacts the rate of healing (Pichardo and van Merkesteyn, 2016; Aljohani et al., 2018; Giudice et al., 2018). In addition, the study was performed at a single healthcare facility, reducing the bias. Last, the modalities of hAM application (side, folding, number, *etc*.) were not described.

Based on Lindenmair’s study (Lindenmair et al., 2010) and Eilbl’s patent (Eibl and Redl, 2011), we considered hAM an innovative medication for bone repair. Meanwhile, we have accumulated extensive experience with hAM and perinatal tissues (Gindraux and Obert, 2010; Obert et al., 2012; Gindraux et al., 2013; Laurent et al., 2014a; Laurent et al., 2014b; Gindraux et al., 2017; Laurent et al., 2017; Centurione et al., 2018; Fénelon et al., 2018; Laurent et al., 2018; Bourgeois et al., 2019; Fenelon et al., 2019; Gualdi et al., 2019; Fenelon et al., 2020; Gulameabasse et al., 2020; Passaretta et al., 2020; Silini et al., 2020; Fénelon et al., 2021a; Odet et al., 2021; Fenelon et al., 2021; Etchebarne et al., 2021; Gindraux, 2021; Dubus et al., 2022a; Dubus et al., 2022b). Because the use of hAM in bone repair still requires some adaptations (Fenelon et al., 2021; Etchebarne et al., 2021), we looked for a disease that could benefit from both wound healing and bone repair as endpoints. MRONJ was that disease.

Thus, we focused on hAM application in oral surgery and established a nomenclature with four theoretical types of hAM surgeries: “implantation,” “apposition,” “whole covering graft material,” and “partial covering graft material” (Odet et al., 2021). Later, we investigated and demonstrated these applications in a pilot study on fresh porcine mandible specimens to assist our clinical practices (Odet et al., 2021).^1^ We noted that hAM suturing was not possible in the MRONJ context, leading us to revise the previous nomenclature. Thus, only two techniques were retained: “hAM implantation with complete coverage” and “hAM implantation with partial coverage” https://youtu.be/GKy3I-n3NRQ.

Here, we did a prospective 6-month pilot study to evaluate the clinical outcome of hAM in terms of wound healing, bone reexposure, pain relief (and thus the quality of life), signs of inflammation, and infection control in patients suffering from stage 2 or stage 3 MRONJ on a compassionate use basis. In addition, we collected potential adverse events and evaluated bone healing or MRONJ recurrence by imaging techniques. Consequently, we describe multiple applications and reapplication of hAM and the use of a collagen sponge to assist hAM surgery, which we believe has not been described in oral surgery, and since the use of hAM in oral surgery is not quite detailed as it is in ophthalmology (Dua and Azuara-Blanco, 1999; Letko et al., 2001; John, 2003), we illustrated different methods of hAM application and we compiled the related difficulties.

## 2 Materials and Methods

### 2.1 Ethical Considerations

This study followed the Declaration of Helsinki on medical protocols and ethics. Patients underwent surgical treatment and follow-up in three different healthcare facilities in France: Besançon University Hospital (Centre Hospitalier Universitaire de Besançon), Reims University Hospital (Centre Hospitalier Universitaire de Reims), and Dijon University Hospital (Centre Hospitalier Universitaire de Dijon).

Each patient had no further therapeutic option available because of impaired health due to their main disease (cancers). Additionally, some patients refused the standard surgical treatment since we could not guarantee the outcome. They were informed of the risks associated with surgical treatment (pain, infection, bleeding, and edema in the short term, as well as possible local complications related to the location and extent of the lesions such as the occurrence of an oroantral communication) and received a detailed description of the procedure. All patients signed an informed consent form prior to surgery and authorized the collection of clinical data and photographic documentation. Ethics committee approval was obtained as part of the clinical randomized trial amniOST.

### 2.2 Study Design and Eligibility Criteria

This study was designed as a prospective 6-month follow-up study for eight patients with stage 2 or 3 MRONJ treated with hAM on a compassionate use basis in the three French hospitals between November 2020 and April 2021. Only stage 2 or 3 MRONJ (AAOMS 2014 classification) patients with surgical treatment failure or bad prognosis due to altered general health were included.

Stage 0 or 1 MRONJ patients or stage 2 or 3 MRONJ patients with no prior surgical treatment were excluded.

Patients were enrolled in a regular prospective follow-up calendar, which consisted of visits on days 7 and 14 and at months 1, 2, 3, and 6. They were either operated on as outpatients or hospitalized the day before and a few days after surgery for immediate postoperative care. Additional recall visits were scheduled, when necessary.

### 2.3 Cohort Characteristics

Clinical data including age, gender, diagnosis, symptoms (pain, acute sinusitis due to local inflammation, infection), general condition, MRONJ stage, and treatment response for MRONJ are reported in Table 1.

**TABLE 1:** Patient and MRONJ data and follow-up.

Patient number	Age	Gender	Centre	Diagnosis	Therapy	MRONJ stage	Symptoms and general condition	VAS	Number of treated lesions					Infection post-surgery	Wound healing 2 weeks post-surgery	Wound healing 1 month post-surgery	Wound healing 3 months post-surgery	Wound healing 6 months post-surgery	MRONJ relapse, bone healing, and/or neoformation 6 months post-surgery	Additional information	
								Preoperative	One week after surgery	Two weeks after surgery	One month after surgery	Six months after surgery								During the follow-up	Reintervention
1	49	M	Besançon	Renal cancer	Subcutaneous denosumab and oral sunitinib	3	Pain, infection, halitosis, bone exposure (nearly 8 cm in left and righ mandible), discontinuation of chemotherapy, malnutrition	9	1	1	0	0	2	No	Bone reexposure (1.5 cm) in sectors 3 and 4 in the premolar area, epithelialization in progress in the posterior parts of the mandible	Persistence of a bilateral 1.5 cm bone exposure in the premolar areas of sectors 3 and 4. Complete epithelialization of the posterior parts of the surgical site	Partial wound healing for the two lesions	Partial wound healing for the two lesions	MRONJ aggravation in sector 4, with mandibular fracture 4.5 months after surgery	Absence of infection. Back to normal nutrition (weight gain +10 kg). Chemotherapy restart. Mandibular fracture 4 months after surgery	No
2	55	F	Besançon	Breast cancer	IV bisphosphonates and subcutaneous denosumab	2	Intense pain, bone exposure in the anterior part of the mandible, infectious episodes	7 (under morphine medication)	2	0	0	0	1 and then the same extended lesion	No	Complete bone reexposure	Persistence of a clean bone exposure	No wound healing for the first application of hAM/Complete wound healing for the second one	Complete wound healing with the second hAM application	Complete bone healing after first surgery on the anterior part of the mandible. Complete bone healing on sector 4 after hAM reapplication	Important tobacco consumption early after surgery. Absence of infection. Discontinuation in morphine medication after surgery	Yes, 5 months after surgery: hAM reapplication on sectors 3 and 4
3	88	M	Dijon	Prostate cancer	Subcutaneous denosumab	2	Bone exposure left mandible, halitosis	1	0	0	0	0	1	No	Complete wound healing, almost complete epithelialization, hAM almost completely resorbed	—	—	Partial wound healing	No radiological examination. Patient still asymptomatic	Visits after 2 weeks missed, the last follow-up at 6 months. Absence of infection. Bone reexposure on the lingual part of the surgical site	No
4	70	F	Dijon	Breast cancer	IV bisphosphonates and subcutaneous denosumab	3	Mandibular osteitis with a cutaneous fistula facing the MRONJ site, 10 cm bone exposure in the anterior sector of the mandible	0	0	0	0	0	1	Administration of antibiotics for weeks (preoperative osteitis)	Complete bone reexposure	Complete bone exposure	No wound healing	No wound healing	Complete bone healing	Absence of infection	Yes, 8 months after surgery: infrahyoid flap
5	71	F	Reims	Breast cancer	Subcutaneous denosumab	3	Pain, 2 cm bone exposures in the anterior part of the mandible, infection with a cutaneous fistula facing the MRONJ site	9	0	0	0	0	1	No	Almost complete epithelialization	Complete epithelialization, surgical site healed	Complete wound healing	Complete wound healing	Stability in MRONJ lesions	Absence of infection. Presence of two painless outgrowths on the anterior part of the mandible 4.5 months after surgery	No
6	69	F	Reims	Breast cancer	IV bisphosphonates and subcutaneous denosumab	2	3 MRONJ lesions: left maxillary (with chronic sinusitis), left and right mandible	2	1	0	0	0	Only left maxillary	No	Epithelialization in progress, minimal bone exposure in the anterior part of the surgical site, hAM not completely resorbed in the posterior part	Complete epithelialization, surgical site healed	Complete wound healing	Complete wound healing	Osseous neoformation on MRONJ site	Absence of infection	No
7	76	F	Reims	Multiple myeloma	IV bisphosphonates	3	Infection, halitosis, 5 cm bone exposure in left mandible	0	0	0	0	0	1	No	Collagen sponge and hAM not visible, wound healing in progress in the anterior part, granulation tissue with bone exposure on the vestibular and lingual sides in the posterior part	Complete healing of the anterior part, persistence of a bone exposure in the posterior part	Partial wound healing	Partial wound healing	Stability in MRONJ lesions	Absence of infection. Lack of oral hygiene after surgery	No
8	62	F	Reims	Multiple myeloma	IV Bisphosphonates	3	Infection, halitosis, 5 cm bone exposure in left mandible	1	0	0	0	0	1	No	Collagen sponge and hAM not visible, wound healing in progress in the posterior part, infracentimetric bone exposure in the anterior part	Healing of the posterior part, persistence of a bone exposure in the anterior part	Partial wound healing	Partial wound healing	Stability in MRONJ lesions	Absence of infection	No

M: Male: F: female; IV: intravenous; SC: subcutaneous; VAS: visual analog scale; hAM: human amniotic membrane.

Two patients were males and six were females. Their age ranged from 49 to 88 years old (mean age: 68 ± 12.2).

As seen in Table 1, patient 1 (P1) suffered from renal cancer and was treated with subcutaneous denosumab and oral sunitinib; P2, P4, P5, and P6 suffered from breast cancer and were treated with IV bisphosphonates and subcutaneous denosumab or subcutaneous denosumab alone; P3 suffered from prostate cancer and was treated with subcutaneous denosumab; P7 and P8 suffered from multiple myeloma and were treated with IV bisphosphonates.

P1, P4, P5, P7, and P8 suffered from stage 3 MRONJ; P2, P3, and P6 from stage 2 MRONJ. P1 had bilateral posterior mandibular MRONJ; thus, both lesions were treated; P2, P4, and P5 had anterior mandibular MRONJ. P3, P7, and P8 had a left mandible (or sector 3) MRONJ. P6 had 3 MRONJ sites in the left maxilla (or sector 2), left mandible, and right mandible (or sector 4). Only his symptomatic sites—left maxilla—were treated.

Before the inclusion in the study, all patients suffered from painful bone exposure, with P2 being treated with morphine. All patients had one or more episodes of infection before surgery. All patients completed a 6-month follow-up.

### 2.4 hAM Preservation and Preparation

The hAM was cryopreserved and obtained from AICT bank from French Blood Center at Besançon (Établissement Français du Sang, EFS), Tissue Bank of Lille University Hospital (Banque de Tissus du CHRU de Lille), and Tissue Bank of Rouen University Hospital (Banque de Tissus CHU de Rouen) (Table 2).

**TABLE 2:** hAM and surgical data.

Patient number	Tissue Bank	hAM size	hAM number	hAM cutting	Difficulties during surgery	Use of a collagen sponge	Water-Tight closure
1	Besançon	4.7 cm diameter disk	3	No	hAM detachment from the nitrocellulose support with forceps, spoiling both hAM and the support, impossibility to orient hAM properly, difficulty to manipulate hAM once detached from the support	No	Yes
2	Besançon	4.7 cm diameter disk	1	No	None	No	Yes
3	Lille	4.7 cm diameter disk	1	No	Difficulties to orient hAM once detached from the support	No	No
4	Rouen	3.0 × 3.0 cm square	3	No	Difficulties to close the wound hermetically without tension	No	Yes
5	Lille	4.7 cm diameter disk	1	No	Difficulties to orient hAM once detached from the support	No	Yes
6	Lille	4.7 cm diameter disk	1	No	None	Yes	No
7	Lille	4.7 cm diameter disk	1	No	None	Yes	No
8	Lille	4.7 cm diameter disk	1	No	None	Yes	No

hAM: human amniotic membrane.

A piece of hAM of 4.7 cm in diameter (from AICT Bank and Tissue Bank of Lille) or a squared 3 × 3 cm piece (from Tissue Bank of Rouen) was transported with dry ice blocks to ensure its cryopreservation at −80°C. Both hAM pieces were stored in glycerol on nitrocellulose support, the epithelial side facing the support. Upon receipt, they were thawed for 2 h at room temperature for AICT bank and 30 min for Tissue Banks of Lille and Rouen. Three 5 min rinses in saline or hypotonic injection solution were done for allografts from AICT bank and one 1 min rinse for those from Tissue Banks of Lille and Rouen.

### 2.5 Surgical Procedures

#### 2.5.1 MRONJ Removal and Tissue Debridement

Surgery was performed either under local or general anesthesia or both to prevent pain upon waking.

After intra- and extraoral disinfection with Povidone-iodine, local injections without vasoconstrictors were made around the MRONJ sites. After crestal incisions on each side of the bone exposure, a full-thickness muco-periosteal flap was raised—at least 2–3 mm—to access bone tissue but with minimal flap length. Bone samples were harvested and sent for bacterial and histological analysis.

Necrotic bone was then removed—completely if possible, by exposing bleeding bone—using a motorized round burr under saline irrigation or by simply performing a sequestrectomy when necessary. The remaining bone was regularized, preserving the patient’s mucosa after suturing and during wound healing. Tissue debridement was also achieved by removing the altered mucosal edges, if necessary.

#### 2.5.2 hAM Handling

**FIGURE 1:**
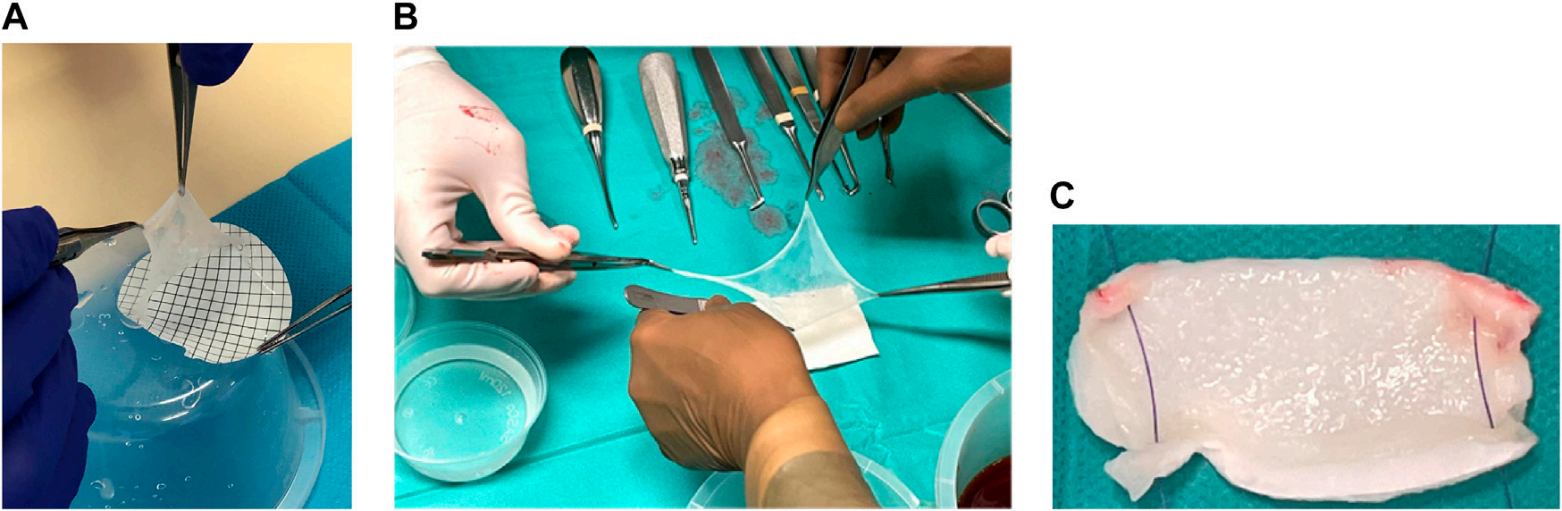
**(A)** hAM detachment from the nitrocellulose support. **(B)** hAM application technique option 2: Two operators applied the hAM flat on the surgical site with “four hands.” **(C)** hAM application technique option 3: hAM was sutured to a collagen sponge (Pangen®, Urgo medical, France).

The hAM was detached from the nitrocellulose support and applied with its mesenchymal side facing the bone and its epithelial side facing the gingiva. Two manipulators were necessary, one to detach the hAM with two forceps while the other held the support with another set of forceps (Figure 1A).

Three options for hAM handling were identified:

- Option 1: on the surgical site, the surgeon held the hAM with the two forceps, while the other unfolded it with two other forceps.
- Option 2: two surgeons applied the hAM flat on the surgical site with “four hands” (Figure 1B).
- Option 3: a resorbable collagen sponge (Pangen®, Urgo Medical, France) was used to supplement hAM handling and application (Figure 1C).

#### 2.5.3 hAM Surgery

hAM and surgical data are summarized in Table 2.

**FIGURE 2:**
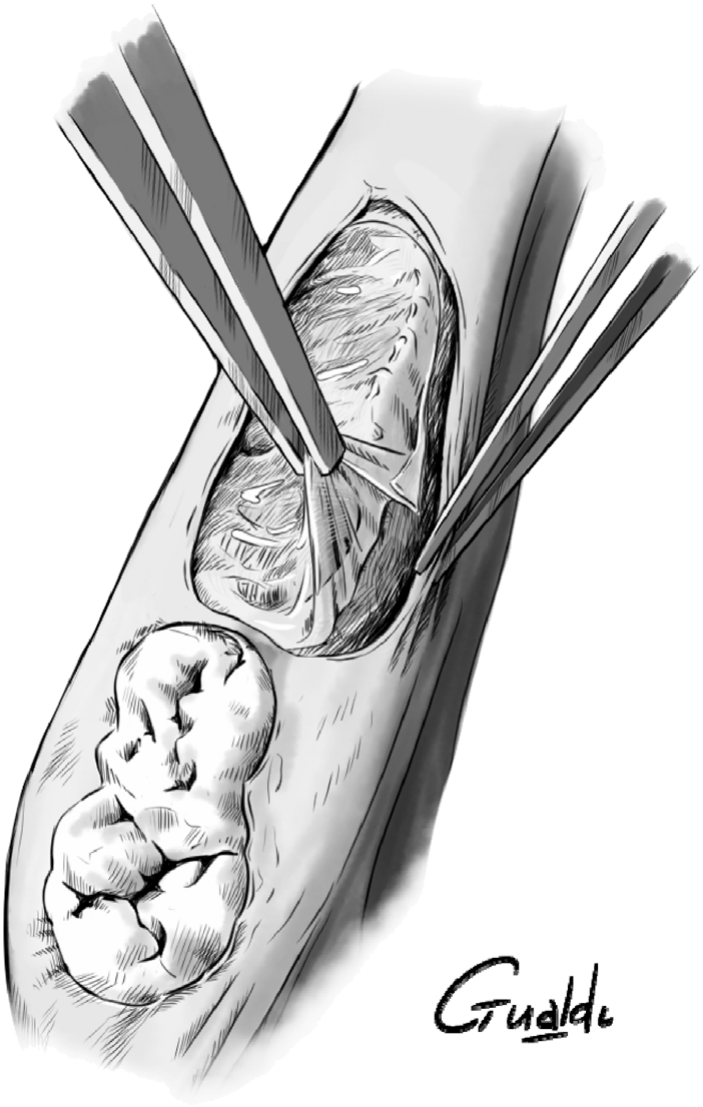
hAM burying between bone and gingiva.

**FIGURE 3:**
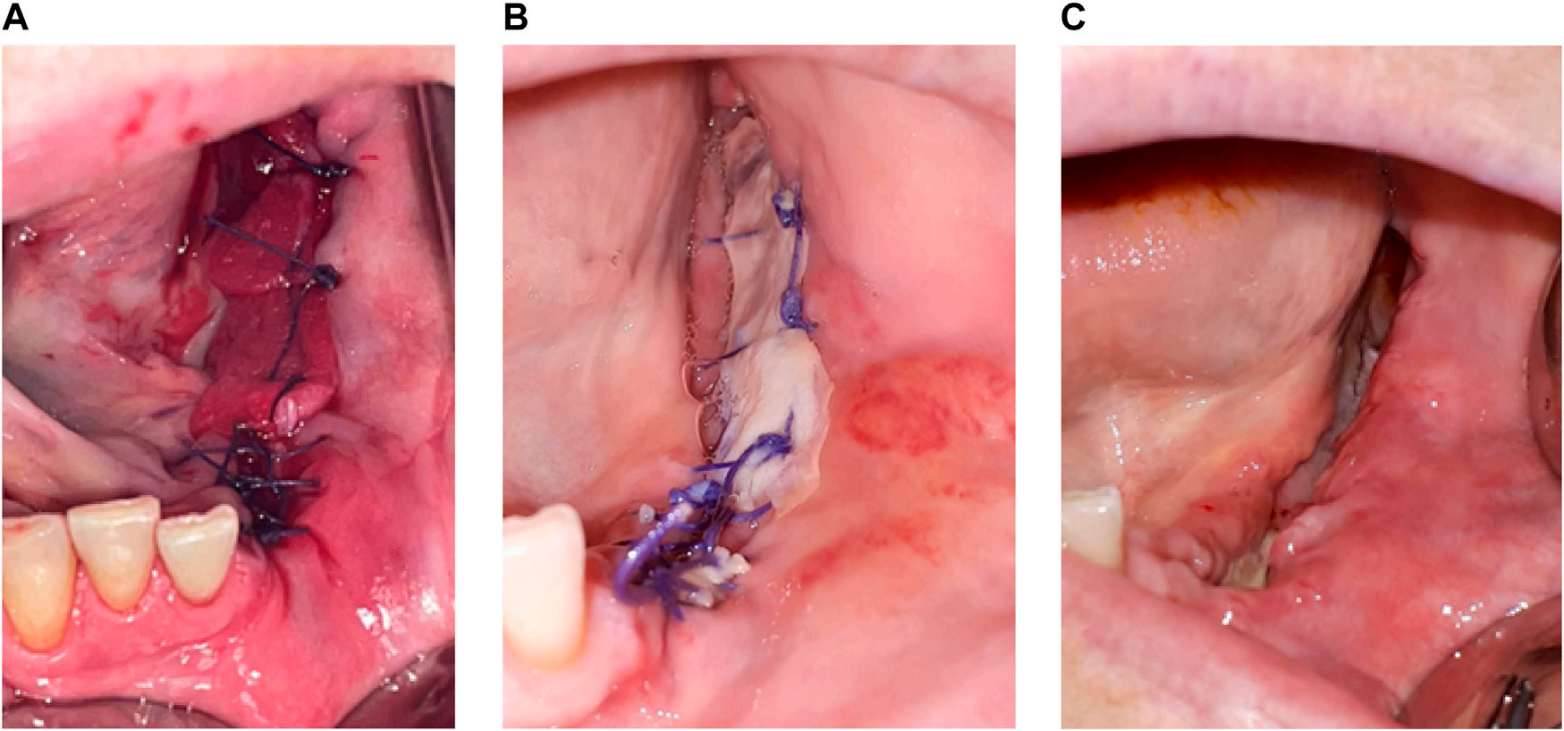
*Patient 8* **(A)** hAM application, sutured on a collagen sponge. **(B)** Three days post-surgery. **(C)** Ten days post-surgery, with the reepithelialization on more than 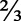
of the surgical site.

In all surgeries, the hAM was tucked between the bone and the mucosa (Figure 2). For P6, P7, and P8, the hAM was applied flat on the collagen sponge and sutured at its ends using resorbable 5/0 Vicryl sutures, with the mesenchymal side facing the sponge, and the epithelial side facing the gingiva (Figure 3A).

The number of hAMs used depended on the defect size. One hAM was applied in P2, P3, and P5 to P8, and three hAMs were applied in P1 and P4. They were applied without being trimmed or cut.

**FIGURE 4:**
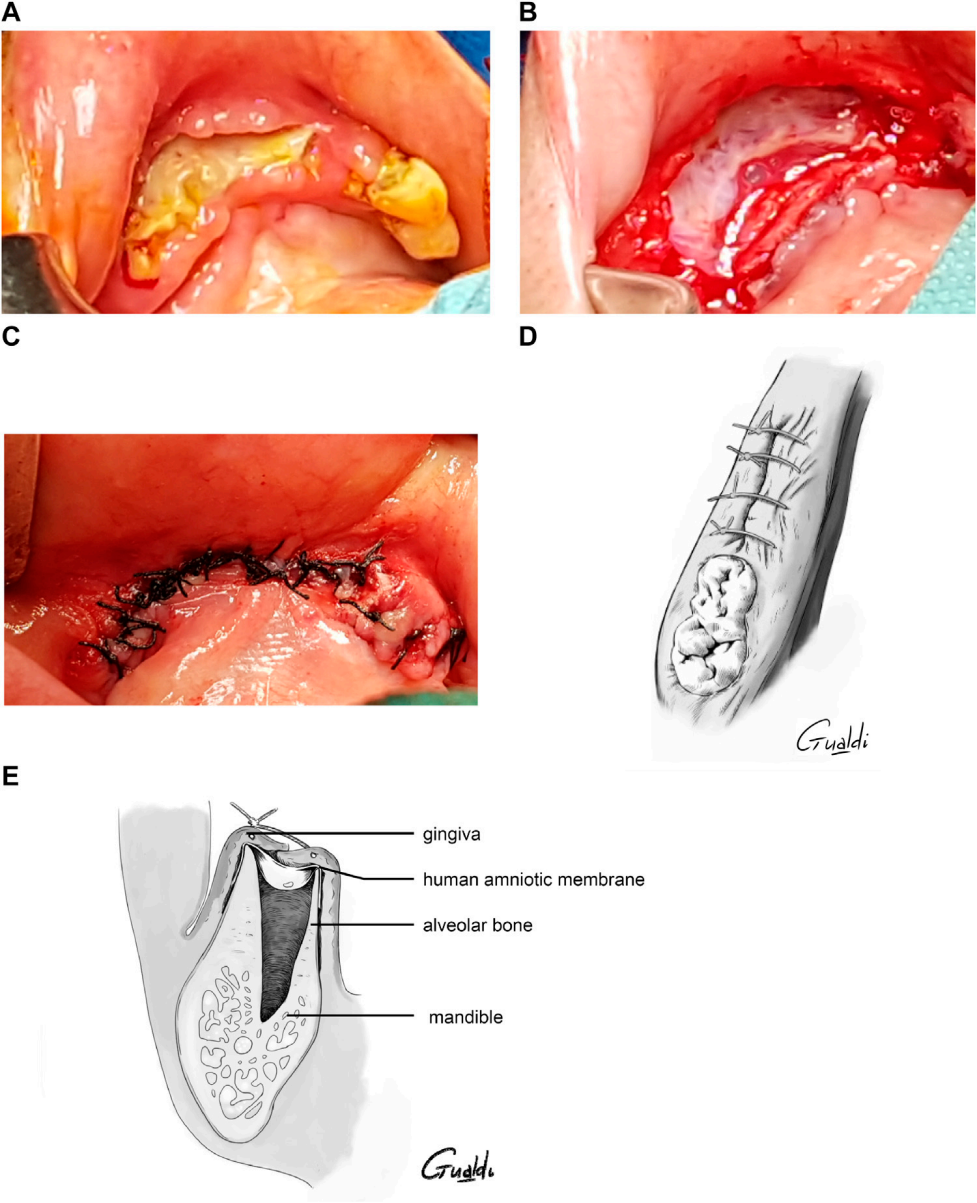
*Patient 2* **(A)** Anterior mandibular stage 2 MRONJ. **(B)** hAM application. **(C)** Hermetical sutures from “hAM implantation with complete coverage” nomenclature (Odet et al., 2021). Here the sutures were done above the implanted hAM which was not visible. **(D)** Upper view and **(E)** Sagittal section illustrations of “hAM implantation with complete coverage” nomenclature.

**FIGURE 5:**
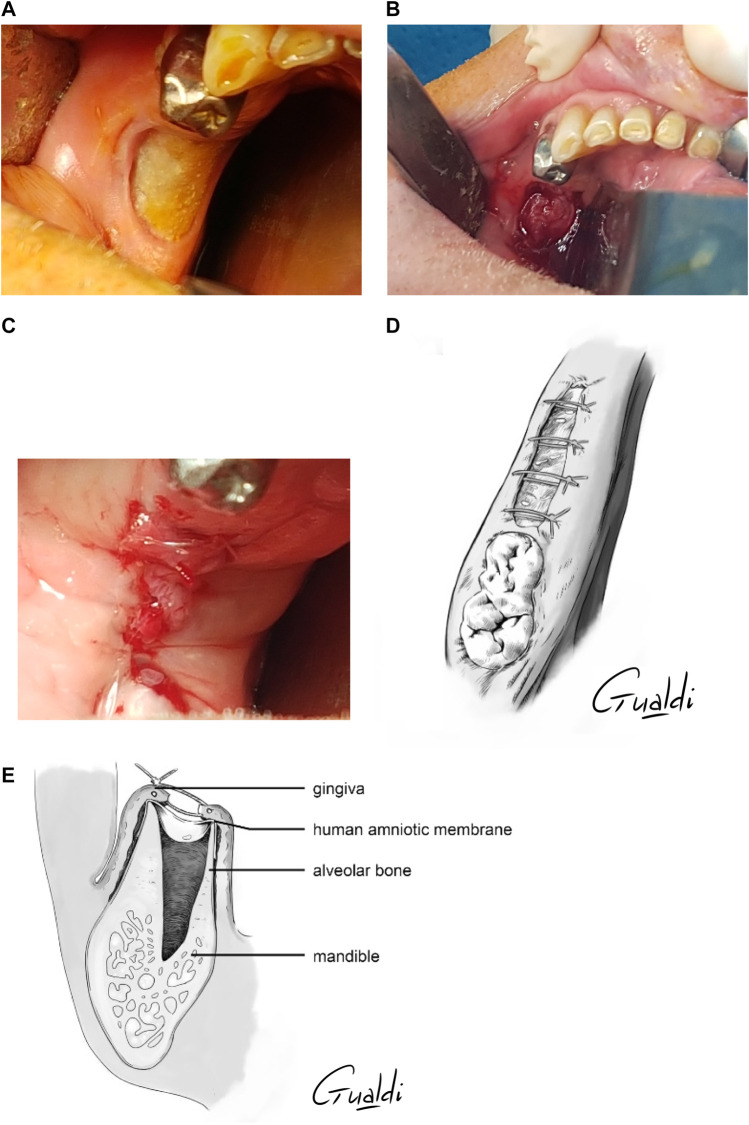
*Patient 3* **(A)** Sector 3 posterior stage 2 MRONJ. **(B)** hAM application. **(C)** Non-hermetic sutures from “hAM implantation with partial coverage” nomenclature (Odet et al., 2021). Here the gingiva was sutured above the hAM, but leaving the hAM exposed in the oral cavity. **(D)** Upper view and **(E)** Sagittal section illustrations of “hAM implantation with partial coverage” nomenclature.

Depending on the quantity of necrotic bone removed, the gingiva was sutured above the hAM as tightly as possible, without tension, leaving it more or less exposed in the oral cavity. Water-tight closure was possible in four cases (P1, P2, P4, and P5) (Figures 4A–E), whereas the hAM was left exposed in the oral cavity in P2, P6, P7, and P8 (Figures 5A–E). In P1 and P5, the hAM sides were hard to define because the hAM was folded upon itself.

### 2.6 Postsurgical Care

Patients had a 2-week antibiotic course prescribed (Amoxicillin + clavulanic acid, 1 g *3/day, and in case of allergies: Clindamycin 600 mg *3/day). P4 had a 6-week-long antibiotic course because of osteitis. Every patient has also been prescribed Chlorhexidine mouth rinses for 10 days.

### 2.7 Clinical Evaluation and Outcome Measures

#### 2.7.1 Bone Reexposure

At each follow-up visit, the primary endpoint was determined through a clinical evaluation of bone reexposure (measured with a soft plastic ruler) and the extent of wound healing.

#### 2.7.2 Pain, Signs of Inflammation, and Infection Control

Secondary endpoints were evaluated at each follow-up visit (Table 1):

- Pain, rated by the patient using a VAS, from 0 to 10 (0 = no pain at all, 10 = worst pain imaginable).
- Signs of inflammation, which were evaluated clinically, with the presence of local erythema and an easily hemorrhagic surgical site.
- Infection, defined as the presence of purulent discharge, abscess, or cellulitis on clinical examination.

#### 2.7.3 Adverse Events

Any adverse events (local or systemic, mild, or serious) were documented throughout the follow-up period: edema, erythema, hematoma, hemorrhage, infection, allergy to the hAM or one of its preservation agents, deep vein thrombosis, pulmonary embolism, upper or lower limb ischemia, and secondary bed rest.

#### 2.7.4 MRONJ Relapse, Bone Healing, and/or New Bone Formation

Orthopantomography was done preoperatively and at 3 and 6 months after surgery coupled with Cone Beam Computed Tomography (CBCT) when necessary to assess MRONJ relapse, bone healing, and/or new bone formation. One patient (P6) underwent Positron emission tomography–computed tomography (PET-CT), prescribed as part of her cancer diagnosis and follow-up.

### 2.8 Statistical Analysis

The Wilcoxon signed-rank test for paired data was applied. Statistical significance was assumed for a *p* value of < 0.05.

## 3 Results

### 3.1 Bone Reexposure and Wound Closure

**FIGURE 6:**
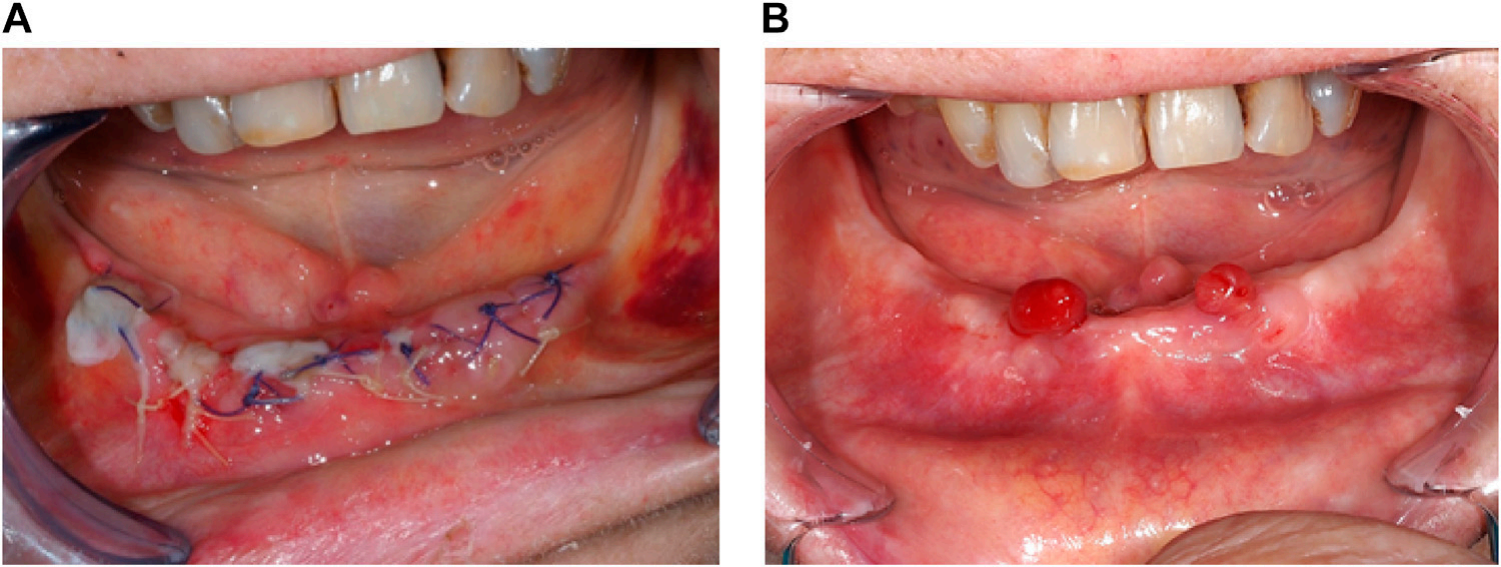
*Patient 5* **(A)** Wound healing beginning at Day 4. **(B)** Two painless and benign granulation tissues 4.5 months post-surgery.

When nonwatertight sutures were made, leaving the hAM exposed in the oral cavity, the allograft started to resorb within the first week postoperatively and was not visible after 2 weeks. As reported in Table 1, complete wound closure was observed in three patients (P3, P5, and P6) 2 weeks after surgery. P3 missed did not attend his postoperative visits past 2 weeks. His last follow-up visit occurred at 6 months postoperatively: he remained asymptomatic with bone reexposure on the lingual part of the surgical site. In P5, wound healing was identifiable from day 4 (Figure 6A); two painless granulation tissue masses appeared at the grafted site 4.5 months after surgery (Figure 6B). These outgrowths were painless, erythematous, and bled easily when touched during clinical examination but did not delay the wearing of a mandibular prosthesis.

Partial bone reexposure was reported in three patients (P1, P7, and P8) 1 week after surgery. In P1, both treated lesions had partial bone reexposure (less than one-third relative to preoperatively) located on the anterior part of the wound. The patient, unfortunately, broke the left horizontal branch of his mandible after 4 months (probably because the remaining bone thickness was less than 1 cm, with only the basilar cortical bone) but remained asymptomatic. We decided not to treat this fracture surgically, only to monitor it. At the 6-month follow-up visit, this fracture remained asymptomatic. In P7, the anterior mandibular part healed properly; the posterior mandibular part healed partially with granulation tissue and no bone exposure in the median part, whereas the lingual and vestibular parts of the alveolar crest were reexposed. In P8, the collagen membrane was still identifiable 3 days after surgery (Figure 3B). Ten days after surgery, the posterior mandibular part had healed correctly, but the bone was still reexposed in the anterior mandibular part (Figure 3C).

Complete wound dehiscence occurred in two patients (P2 and P4) 1 week after surgery. However, these patients remained asymptomatic. P2 (who had wound dehiscence in the anterior sector of the mandible) later suffered from a painful bone reexposure in the premolar area in right mandibular, with symptoms of an infection. Thus, 5 months after the first application, hAM was reapplied on the reexposed lesion combined with a single hAM application on the new MRONJ lesion in the right mandibular. The whole surgical site was closed tightly. On day 7, partial bone reexposure was noticed in the premolar area. After 1 month, the whole surgical site was almost completely reepithelialized. After 3 months, the patient was completely pain-free, the surgical site was clean, and no adverse effect was noticed.

At both 3 and 6 months, complete wound healing was observed for P2 (who had undergone hAM reapplication), P5, and P6. Partial wound healing was observed in P1, P3 (at his unique follow-up at 6 months), P7, and P8. P4 did not show signs of wound healing, only bone healing at 6 months, evaluated radiologically.

### 3.2 Pain and Infection

Before surgery, the mean VAS pain score was 3.5/10 (±3.61 SD) with one patient being treated with morphine (Table 1). All patients had persistent chronic pain, and out of eight patients, four had a score >2 and six had a score >1. The use of hAM in this context was able to significantly reduce the pain perception between the preoperative measure and the 1-week postsurgery measure (*p =* 0.03). The score decreased for all of them, with the largest decrease being from 9 to 1 for P1. Pain relief was complete (0/10) for all patients at 1 month. At 6 months, P3 did not report having any pain during the follow-up visits he missed.

As the first example of high pain levels, P1, who suffered from renal cancer, evaluated his pain at 8 to 9/10 on VAS, seriously altering his quality of life. He was fed by a nasogastric tube and suffered from malnutrition, leading to the discontinuation of his chemotherapy treatment. He experienced immediate pain relief after surgery, with a 0/10 score on VAS. Even though he had a 1 cm long wound dehiscence on both sides, his nasogastric tube was taken out after 3 weeks. He was then able to eat a soft-food diet, which he had not been able to do for 1 year. He gained 10 kg in 2 months and was able to resume chemotherapy for his renal cancer. At the 6-month follow-up visit, even with his mandible fracture, the patient remained pain-free and the surgical sites (MRONJ sites in left and right mandibular sectors) were uninfected.

As a second example, P2, who was suffering from breast cancer, evaluated her pain at 7/10 on VAS despite being treated with morphine. One week after surgery, she was completely asymptomatic and had ceased her morphine medication. As above, 3 months after hAM reapplication, the patient was completely pain-free.

As a third example, P5 evaluated her pain intensity at 7/10 and suffered from multiple infection episodes with cellulitis and oral fistulas with purulent discharge, causing halitosis. She had immediate pain relief after surgery and had no pain 1 week later. She did not experience any infections afterward. As shown above, the two outgrowths were painless and benign without impacting her dental prosthesis wear and allowing her to resume a normal diet.

Besides pain relief, no symptoms of infection were noticed during the whole follow-up in all patients.

### 3.3 Local Inflammation and Adverse Events

In all patients and even after multiple hAM applications or reapplication, no inflammatory symptoms and no erythema were noticed by the surgeons at the surgical sites early after surgery; also, the wound was not hemorrhagic (Table 1). No adverse events were reported. Painless, erythematous, and easily hemorrhagic outgrowths spontaneously appeared in P5’s mouth but we were unable to attribute these tissue extensions to the hAM application in an inflammatory and infectious disease such as MRONJ.

### 3.4 MRONJ Relapse, Bone Healing, and/or New Bone Formation

**FIGURE 7:**
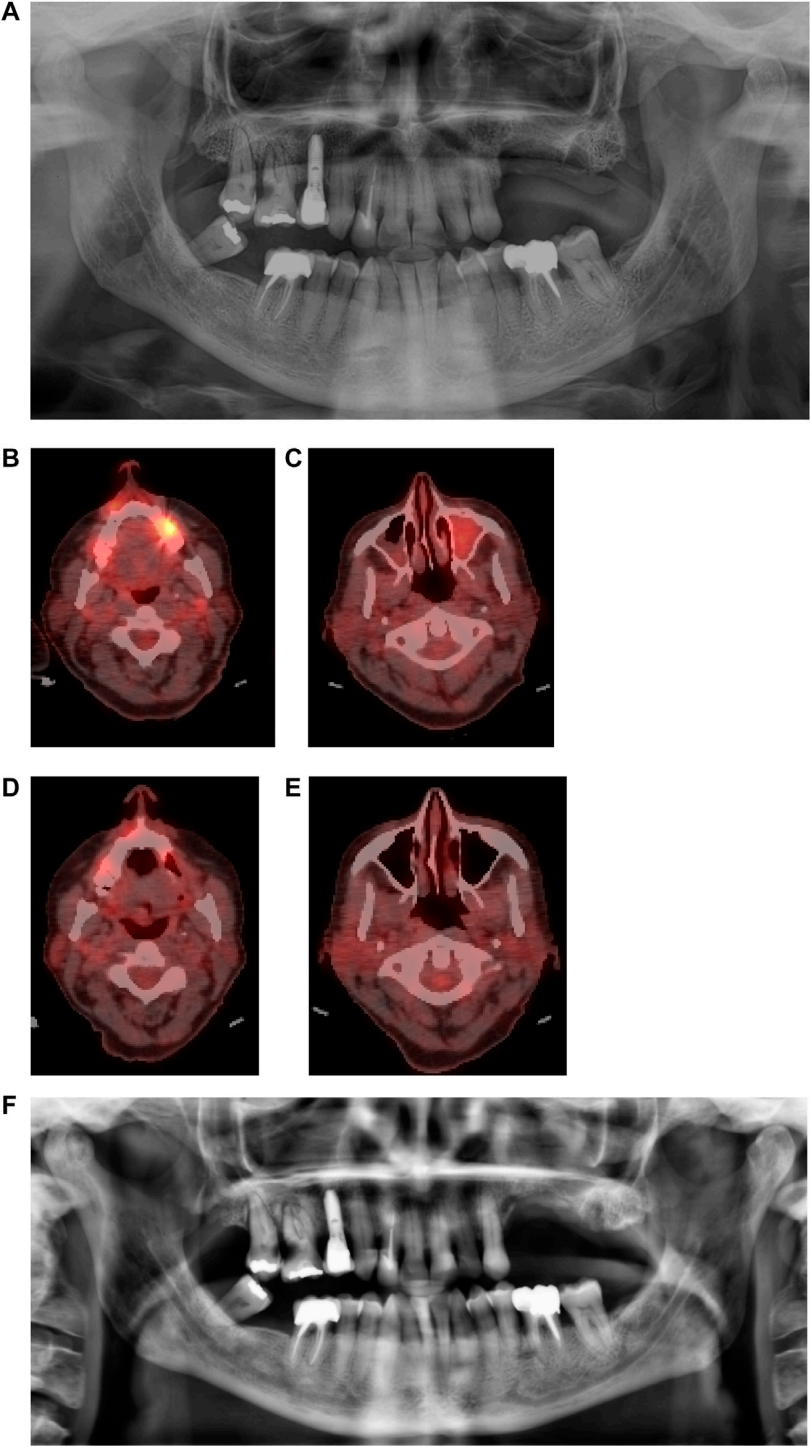
*Patient 6* **(A)** Pre-operative orthopantomography (OPT) showing a stage 2 sector 2 MRONJ; **(B,C)** Positron-Emission Tomography (PET) before surgery, showing a hypermetabolic signal regarding the MRONJ site in sector 2, and an inflammatory left maxillary sinus; **(D,E)** Three months post-operative PET, with a considerable decrease of the hypermetabolic signal and a healthy left maxillary sinus. **(F)** Six months post-operative OPT with osseous neoformation in sector 2.

In five cases (P2, P4, P6, P7, and P8), the bone healed completely without MRONJ recurrence, with or without complete reepithelialization above the surgical site (Table 1). In P2, 5 months after the first hAM application, complete bone healing was observed on the anterior part of the mandible, and 6 months after the hAM reapplication, complete bone healing was observed in the right mandibular. As another example, P6 had a preoperative, 3-month, and 6-month postoperative PET-CT. The preoperative examination showed a hypermetabolic signal at the MRONJ site in the left maxilla, with an inflammatory and infected left maxillary sinus (Figures 7A–C). Three months after surgery, this hypermetabolic signal completely disappeared, and the left maxillary sinus was no longer infected (Figures 7D,E). The 6-month postoperative orthopantomography showed new bone formation (Figure 7F). Since this patient was asymptomatic, no further surgical treatment was performed.

Bone healing was incomplete in two patients. P1, who had an extensive preoperative MRONJ, had an asymptomatic relapse of MRONJ in the left and right mandible after 3 months, with a mandibular fracture after 4.5 months documented by CBCT. In P5, the MRONJ site was extensive and minimum bone resection was possible. Postoperative imaging showed that the MRONJ had stabilized 6 months after surgery. P3 did not have any radiological examinations.

## 4 Discussion

Our study evaluated the benefits of using hAM in MRONJ on wound healing, quality of life (pain relief and infection control in our study), and signs of inflammation. Here, the application of hAM in patients who have exhausted all treatment options offered immediate pain relief and improved quality of life. Due to the clinical status of these patients, these first observations were already a positive result beyond the management of the lesions.

Among the eight patients treated in the context of compassionate use, three patients had a complete closure of the surgical site with proper reepithelialization at 2 weeks. Epithelialization started 1 week after surgery and was complete 1 month after, consistent with observations in other oral surgery indications (Odet et al., 2021). As one of these three patients missed several follow-up visits, the complete healing rate at 1 month (2 patients out of 7: 28.6%) was lower than in Ragazzo’s cohort (78%) at the same time point (Ragazzo et al., 2021). Also, three asymptomatic patients had partial wound dehiscence 1 week after surgery and partial healing 3 months later.

In our study, complete healing at 3 months was found in 25% (2 patients out of 8) or 33% (3 lesions out of 9) of the subjects, whereas partial healing was found in 37.5% (3 patients out of 8) or 44% (4 lesions out of 9) of the subjects. At 6 months, thanks to hAM reapplication in P2, 80% of the lesions had complete or partial wound healing (30 and 50%, respectively) while 62.5% of patients were in stage 3. Our multicenter study was carried out in the context of compassionate use. Indeed, there are few surgical treatment options for MRONJ management, other than extensive flaps covering the surgical site. These flaps were too invasive for patients with impaired health. Additionally, these patients had a low healing capacity because of their cancer, had received multiple IV courses of chemotherapy, and mainly had stage 3 MRONJ (62.5%). On the contrary, Ragazzo’s cohort was done at a single hospital and combined 1) all stages with a different SIPMO/SICMF classification (eight patients in stage 1, 13 patients in stage 2, and two patients in stage 3), 2) various diagnoses (cancer and osteoporosis), and 3) various routes of drug administration (per os, IV and subcutaneously) and type of drugs (bone antiresorptive agents and angiogenesis inhibitors). However, it is widely accepted that healing is easier for stage 1 and stage 2 MRONJ patients suffering from osteoporosis treated with per os bone antiresorptive drugs (Pichardo and van Merkesteyn, 2016; Aljohani et al., 2018; Giudice et al., 2018). Similarly, a small single-center cohort study (*N* = 5) has reported complete mucosal coverage with a healing rate of 80% at 3 months in stage 2 MRONJ cases, which are easier to heal (Canakci et al., 2021). Two asymptomatic patients had complete wound dehiscence 1 week after surgery. One was probably due to a fragile and very low-quality gingiva (due to chronic infection and inflammation) and to resumption of smoking right after the surgery (patient 2). In this case, another necrotic site was noted during a follow-up visit in the right mandible. Since this site was painful, we decided to remove the necrotic bone and to reapply hAM on the whole area of exposed bone. After this reapplication, complete wound epithelialization was noted after 1 month and maintained over time. In patient 4, the wound dehiscence was probably related to a mechanical issue (excessive tension on the gingival sutures) and to the location of the MRONJ lesion (on the lingual part of the alveolar crest where tongue movements could hinder wound healing). Bone healing was achieved for this last patient at 6 months.

Complete wound reepithelialization is linked to bone healing. In our study, the bone lesions were stable in 71% of the patients (5 out of 7 patients); new bone formation was observed in one patient (14%) and the MRONJ got worse in one patient. The total of bone lesion stability and new bone formation (86%) was similar to the partial and complete wound healing rate of MRONJ lesions (80%) at the same time point.

Multiple studies have evaluated bone healing, particularly in MRONJ. The main imaging tools used for both diagnosis and follow-up are orthopantomography and CBCT (Falco et al., 2021; Bouland et al., 2021; Canakci et al., 2021; Ragazzo et al., 2021). Here, we report the use of PET-CT to follow bone repair in one patient who was undergoing this exam for the monitoring of her cancer. The main advantage of PET-CT is that it provides a map of the level of metabolic activity, which can be used at a surgical site and its surrounding structures (Fleisher et al., 2016). In addition, the use of single-photon emission computed tomography (SPECT) has also been reported in the literature and seems to detect metabolic changes as well, whereas CBCT seems to be more accurate in detecting morphological changes (Malina-Altzinger et al., 2019. Çanakçi is the only one who provided detailed postoperative radiological findings after hAM application, with three patients having no extension of bone destruction on both orthopantomography and CBCT after 6 months (Canakci et al., 2021). The radiological findings of the two remaining cases were not provided. Histology has been used to evaluate postextractive alveolus healing following the procedure for socket preservation in a patient receiving oral Bisphosphonates for more than 6 years (Falco et al., 2021). However, like the American Association of Oral and Maxillofacial Surgeons (AAOMS) recommendations (Ruggiero et al., 2014), we agree that an osseous or mucosal biopsy to follow the wound healing process in MRONJ patients is too invasive and could impact the healing process or reinfect the treated lesions.

Addressing persistent and chronic pain is one of the major goals of MRONJ management. hAM has been widely described as being able to reduce the pain experienced by patients (Mamede et al., 2012). It acts as a biological dressing that protects the exposed nerve (Farhadihosseinabadi et al., 2018) and has analgesic effects often reported in the oral surgery field (Odet et al., 2021). In our cohort, all patients had persistent and chronic pain. All were significantly asymptomatic in terms of pain and infection symptoms during the entire follow-up period. Remarkably, pain relief was obtained quickly after surgery, improving quality of life at the same time. Normal nutrition and, when necessary, chemotherapy could be restarted. Our results are consistent with those reported by Ragazzo et al. (2021), where 25 out of 26 patients had complete pain relief at 7 days postoperatively. Additionally, they reported that pain at rest and quality of life improved significantly in the hAM group compared to the resection surgery group.

Infection, often associated with pain and quality of life, is another big challenge in MRONJ management. hAM exerts an antimicrobial effect and therefore protects the wound from infection (Thomas, 2013). It expresses natural antimicrobial molecules such as β-defensins and elafin (King et al., 2007) and has an inhibitory effect against several bacteria (group A *Streptococcus* or *Staphylococcus aureus*) (Kjaergaard et al., 2001), resulting in an antibacterial effect. Its close adherence to the wound surface may also help to prevent contamination (Diaz-Prado et al., 2009). This close adhesion is also known to maintain a moist environment, which contributes to the pain-relieving effect of hAM (Jirsova and Jones, 2017).

hAM is a suitable option for postsurgery applications in wound healing, burn injuries, and ophthalmology (Dadkhah Tehrani et al., 2020) as bacterial infection and biofilm growth are common in these sites (Chen et al., 2012; Mamede et al., 2012). In the last years, our basic knowledge of the antimicrobial activity of fetal membranes has increased (Ramuta et al., 2021) with strong evidence in periodontal disease (Ashraf et al., 2019). Compared to the Ragazzo study (2021), we monitored for infection and reported no infection episodes during the entire follow-up period in our patients.

hAM has low immunogenicity (Kubo et al., 2001), which makes it suitable as an allograft. In our cohort, no matter how many hAMs were applied, no sign of inflammation—monitored by the presence of erythema as well as a tendency for the surgical site to be hemorrhagic—was observed. For patient 5, the outgrowths that spontaneously appeared could either be granulation tissue, due to probable subgingival inflammation, which is easily hemorrhagic clinically, or a botriomycoma, a benign vascular tumor. We were unable to attribute these outgrowths to the hAM application in the context of an inflammatory and infectious disease such as MRONJ. This patient’s follow-up was reassuring. Confident with hAM grafting and the absence of adverse events, we experimented for the first hAM reapplication in the same patient, on the initial lesion whose first application had failed and on a new one. As mentioned in a prior study (Odet et al., 2021), hAM size, number, and reapplication are essential information about the product’s pharmacology. Here, neither hAM number nor reapplication induced any sign of inflammation or adverse events, and its reapplication seems to have a dose effect. Çanakçi et al. also reported successful results in two patients who had two MRONJ lesions treated with hAM (Canakci et al., 2021).

The second goal of our study was to describe in detail and illustrate, for the first time in the literature, our hAM application method and its related difficulties. Here, from a technical point of view, the hAM was difficult to detach from its nitrocellulose support. Ophthalmologists are assisted by a microscope and blunt forceps. For oral surgery, two surgeons and blunt forceps were necessary. Three methods of hAM handling were identified. In the first one, the hAM’s orientation was not easy to preserve because the hAM had folded on itself. The “four hands application technique” or a collagen sponge solved the problem. Therefore, we reported for the first time in literature the innovative use of a collagen sponge to improve hAM handling, orientation, and protection in the oral cavity.

Once applied, it was easy to tuck the hAM between the bone and mucosa, as it was remarkably adherent to the gingiva sutured above it. Indeed, we never sutured the hAM directly to the adjacent mucosa, due to the hAM’s fragility. In four cases, the mucosa closure was water-tight. When not possible, less water-tight sutures without tension were applied, leaving the hAM exposed in the oral cavity or protected by the collagen sponge. In this case, clinical hAM resorption was observed and occurred after 7–10 days, as is typical in other oral surgery cases (Odet et al., 2021).

Here, we confirmed that “hAM implantation with complete coverage” and “hAM implantation with partial coverage” are the most suitable techniques for MRONJ surgery as already identified in our pilot study on porcine mandible specimens (Odet et al., submitted). From the four theoretical types of surgery established in our previous nomenclature (Odet et al., 2021), the apposition technique—where the hAM is applied without burying or suturing, left exposed in the mouth and stabilized by any means (cross stitches, pressure dressing, palatal plates, etc.,)—is feasible when gingival suturing or MRONJ lesion localization are inadequate, as occurred with patient 4. The other techniques that involve hAM suturing do not seem to be suitable for MRONJ management.

Complementary treatments to surgery, such as autologous platelet concentrates (APCs), have been developed to improve the healing of bone tissue and reduce recurrence. Like hAM, APCs release growth factors such as vascular endothelial growth factor, epidermal growth factor, platelet-derived growth factors, basic fibroblast growth factor, and transforming growth factor b1. Among APCs, platelet-rich fibrin (PRF), which is simple and inexpensive to prepare, was used to treat MRONJ after bone resection with questionable conclusions (Giudice et al., 2018). Its application, although not described in detail in the literature, is like hAM. Studies have evaluated mucosal integrity, absence of infection, and pain relief and showed a significant difference between the two groups in favor of PRF only at 1 month (*p* < 0.05), whereas no differences were found at 6 and 12 months (*p* > 0.05) (Giudice et al., 2018). Recently, Fortunato et al. reported in a systematic review that APC treatment produced a complete response in 302 lesions (88%), partial response in 23 lesions (6.7%), and negative response in 19 lesions (5.5%), although the clinical evaluations performed at the follow-up visits varied in their time points (1–94 months). The outcome of surgery alone as a treatment was a complete response in 37 lesions (64%), partial response in 14 lesions (24%), and negative response in seven lesions (12%). These findings are not sufficient to establish the effectiveness of APCs in the prevention and treatment of MRONJ. The authors concluded that randomized controlled trials with a large sample size are needed.

The limits of the PRF compared to hAM are 1) its autologous origin so nonqualifiable potential, 2) its nonelastic properties, and 3) the lack of suturing potential. To this end, a case report has described how to improve its potential with an adipose-tissue stromal vascular fraction containing mesenchymal stromal cells and endothelial progenitor cells (Bouland et al., 2021).

hAM is an allogenic tissue with regenerative components through growth factors and stem cells which possess the required properties for MRONJ management in line with pain relief, infection control, and wound healing. In addition, its route of application and resorbability seems to be more consistent when compared to PRF.

## 5 Conclusion

hAM seems to be a promising alternative for MRONJ surgical treatment. It enhances epithelialization, as six of our eight patients showed at least partial wound healing, with granulation tissue or actual gingiva covering the operated site. The patients had significant relief from pain and infectious symptoms, drastically improving their quality of life, and being able to eat again or resume chemotherapy. We detailed and illustrated the surgical application modalities and the obstacles that could be circumvented through the use of a collagen sponge, for example. We did not identify any barriers to the application of multiple hAM patches at the same time and their reapplication in one patient. This very good tolerance suggests that hAM transplantation should be employed as soon as possible without waiting for patients to reach stage 3, in order to increase the likelihood of a complete cure. In addition, reapplication seems to be a good option for the most affected patients, if required. This study will be soon followed by a multicenter randomized controlled trial in five centers in France (grant PHRCI-2020).

## Data Availability

The original contributions presented in the study are included in the article/Supplementary Material; further inquiries can be directed to the corresponding author.

## Footnotes

1 Odet, S., Meyer, C., Solecki, L., Weber, E., Chatelain, B., Euvrard, E., et al. (2021). Human Amniotic Membrane Application in Oral Surgery - an *Ex Vivo* Pilot Study. Front. Bioeng. Biotech. submitted manuscript.
