# Erratum: Tips and tricks and clinical outcome of cryopreserved human amniotic membrane application for the management of medication- related osteonecrosis of the jaw (MRONJ): A pilot study

**DOI:** 10.3389/fbioe.2022.1058241

**Published:** 2022-11-03

**Authors:** Frontiers Production Office

**Keywords:** human amniotic membrane, osteonecrosis, oral mucosa, allograft, bisphosphonates, denosumab, antiangiogenic drugs

Due to a production error, there was a mismatch in Figures 4D, 5D, 5E as published. The corrected figures appear below.

Additionally, the inactive video link in the **Introduction** section (paragraph 7) has been replaced with the following link: https://youtu.be/GKy3I-n3NRQ.

**FIGURE 4:**
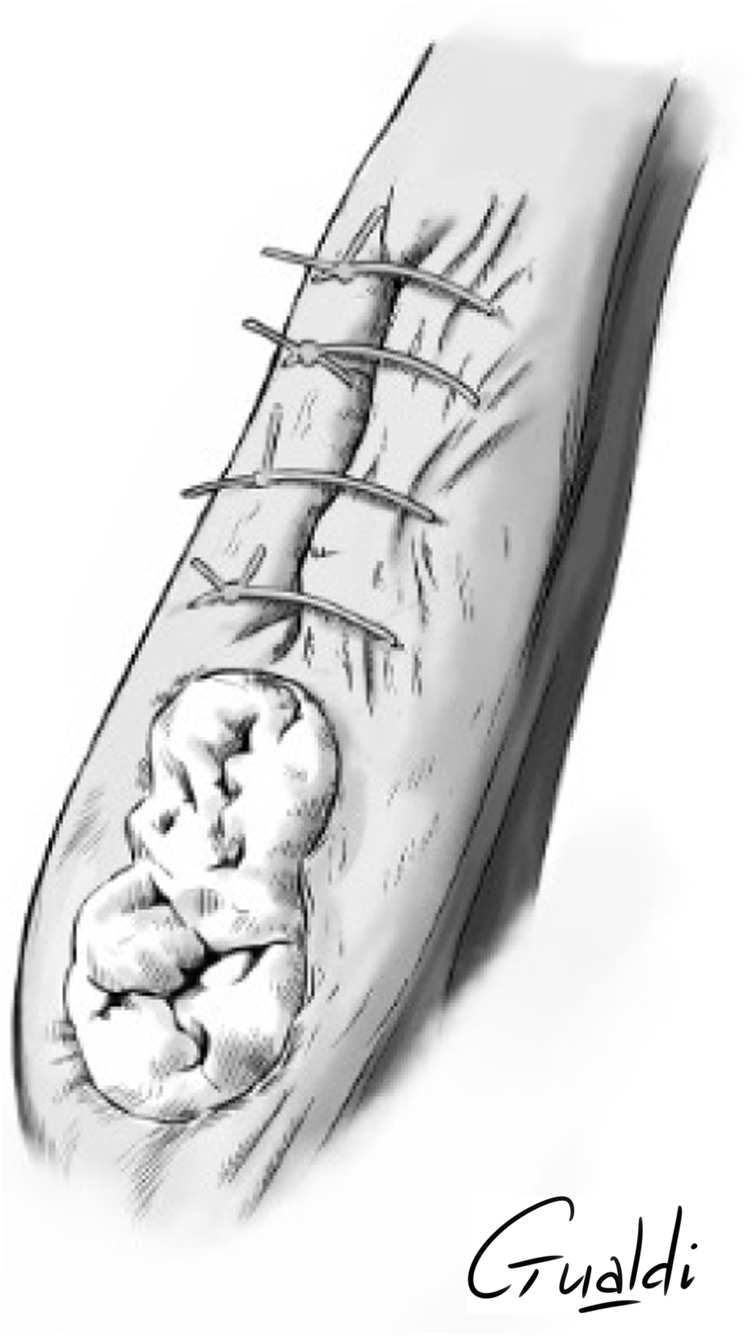
Patient 2 **(A)** Anterior mandibular stage 2 MRONJ. **(B)** hAM application. **(C)** Hermetical sutures from “hAM implantation with complete coverage” nomenclature (**Odet et al., 2021**). Here the sutures were done above the implanted hAM which was not visible. **(D)** Upper view and **(E)** Sagittal section illustrations of “hAM implantation with complete coverage” nomenclature.

**FIGURE 5:**
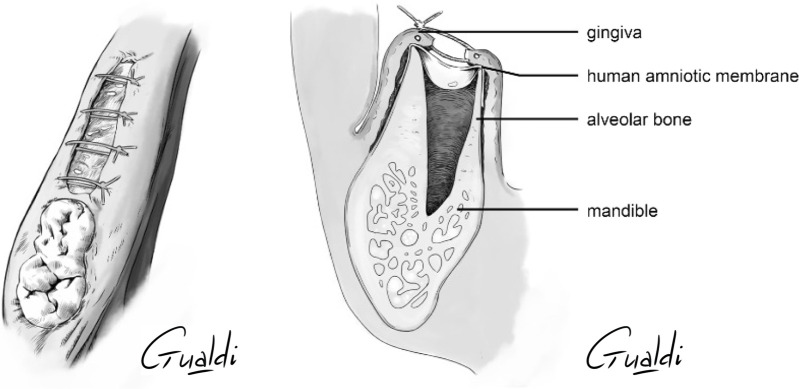
Patient 3 **(A)** Sector 3 posterior stage 2 MRONJ. **(B)** hAM application. **(C)** Non-hermetic sutures from “hAM implantation with partial coverage” nomenclature (**Odet et al., 2021**). Here the gingiva was sutured above the hAM, but leaving the hAM exposed in the oral cavity. **(D)** Upper view and **(E)** Sagittal section illustrations of “hAM implantation with partial coverage” nomenclature.

There were two typing errors in the **Figure 3** legend, the correction appears below:

**FIGURE 3**

Patient 8 (A) hAM application, sutured on a collagen sponge. (B) Three days post-surgery. (C) Ten days post-surgery, with the reepithelialization on more than 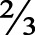
of the surgical site.

The publisher apologizes for this mistake. The original version of this article has been updated.

## Publisher’s note

All claims expressed in this article are solely those of the authors and do not necessarily represent those of their affiliated organizations, or those of the publisher, the editors and the reviewers. Any product that may be evaluated in this article, or claim that may be made by its manufacturer, is not guaranteed or endorsed by the publisher.

